# Advances in MRI of glymphatic system dysfunction associated with obstructive sleep apnea and enlarged perivascular spaces

**DOI:** 10.3389/fneur.2026.1868105

**Published:** 2026-09-10

**Authors:** Chunxue Di, Yue Li, Guangwei Jin

**Affiliations:** 1 North China University of Science and Technology, Tangshan, China; 2 Department of Radiology, East Campus of China Emergency General Hospital, Beijing, China; 3 Department of Medical Imaging, Fangshan Hospital Beijing University of Chinese Medicine, Beijing, China; 4 Department of Otolaryngology, China Emergency General Hospital, Beijing, China

**Keywords:** diffusion tensor imaging analysis along the perivascular space, enlarged perivascular spaces, glymphatic system, magnetic resonance imaging, obstructive sleep apnea

## Abstract

Obstructive sleep apnea (OSA) is closely associated with an increased risk of cerebral small-vessel disease and cognitive impairment. OSA not only impairs cognitive function but also promotes the occurrence and development of enlarged perivascular spaces (EPVS) by impairing glymphatic system (GS) function. Perivascular spaces (PVS) are an important imaging marker of cerebral small-vessel disease and a morphological indicator of the functional status of the GS. However, the pathogenesis of OSA-related EPVS remains unclear, with GS dysfunction considered to be at its core. Magnetic resonance imaging (MRI) is a key tool for evaluating the association between OSA and EPVS, and emerging MRI techniques have provided imaging evidence to reveal the potential link between OSA-related GS dysfunction and EPVS formation. This article reviews the relationship between OSA severity, sleep structure, and the distribution of EPVS and focuses on the application of structural MRI, voxel-based morphometry, diffusion tensor imaging, diffusion tensor imaging analysis along the perivascular spaces, diffusion kurtosis imaging, dynamic contrast-enhanced MRI, phase-contrast and 4D flow MRI, and blood oxygen level-dependent functional MRI in the study of the relationship between OSA and EPVS.

## 1 Introduction

With the intensification of global aging and increasing work and life pressures, sleep disorders, such as sleep apnea syndrome and insomnia, and diseases such as cerebral small-vessel disease (CSVD), are becoming increasingly common. These diseases have become serious public health concerns worldwide. Approximately 1 billion people aged 30–69 years worldwide suffer from obstructive sleep apnea (OSA), with the prevalence of mild OSA ranging from 7.8 to 77.2% in different regions, and that of moderate-to-severe OSA ranging from 4.8 to 36.6%, with prevalence increasing annually (1). Studies have shown that the prevalence of moderate and severe EPVS in low- and middle-income countries is 25.0% in community-dwelling populations (2). OSA is considered an important risk factor for asymptomatic cerebrovascular disease, stroke, and dementia (3). The perivascular spaces (PVS) as an imaging marker of CSVD have been proposed to represent a core anatomical pathway of the glymphatic system (GS) in recent years (4, 5). The main function of the GS is to clear metabolic waste in the brain. Enlarged PVS (EPVS) are influenced by multiple vascular, metabolic, and lifestyle-related factors, including age, hypertension (especially elevated systolic blood pressure), arteriosclerosis, smoking, reduced sleep efficiency, insulin resistance, prolonged course of chronic obstructive pulmonary disease, and hyperhomocysteinemia. These factors may synergistically promote EPVS through blood–brain barrier (BBB) damage, reduced vascular pulsatility, inflammatory responses, and impaired cerebral interstitial fluid drainage (6). Furthermore, OSA has been associated with EPVS, and obesity and BMI are important confounding factors in this relationship. To assess whether OSA independently affects EPVS, it is essential to control for obesity in the analysis. Available studies have demonstrated that the association between OSA and EPVS remains significant after adjustment for BMI (7), suggesting that this association is not solely attributable to obesity. OSA may promote EPVS by affecting sleep structure and impairing GS function (8). Before discussing this mechanism, it is necessary to clarify whether the GS is distinct from the meningeal lymphatic system. The GS refers to the fluid transport network formed by the PVS within the brain parenchyma, whereas the meningeal lymphatic system consists of lymphatic vessels located in the dura mater. Anatomically, the two systems belong to separate compartments; functionally, they are functionally interconnected and jointly contribute to the clearance of metabolic waste from the brain. Currently, the relationship between OSA and white matter hyperintensities is relatively clear, whereas the relationship between OSA and EPVS has been less well studied (9–11). Previous studies have found that the distribution of EPVS is associated with the disease severity and sleep structure of patients with OSA. Compared with conventional magnetic resonance imaging (MRI), the application of advanced MRI techniques to explore the relationship between OSA and EPVS may help provide comprehensive and objective imaging evidence for earlier identification of OSA-related abnormalities, which is crucial to enhance our understanding of the mechanisms underlying the association between OSA and EPVS. This article aims to provide a narratively review of the research progress on the relationship between OSA and EPVS, focusing on summarizing the application value of different MRI techniques in exploring this relationship and delving into the core mechanistic role of GS dysfunction to provide a new imaging perspective for early identification and intervention of small-vessel disease associated with OSA.

## 2 OSA and its main risk factors

OSA is a common sleep-breathing disorder characterized by repeated partial or complete blockage of the upper airway during nighttime sleep despite breathing efforts. During these events, the airflow can be intermittently reduced (i.e., hypopnea) or completely stopped (i.e., apnea) (12). Disease severity is quantitatively assessed using the apnea-hypopnea index (AHI), which is the total number of apnea and hypopnea events per hour of sleep. Based on the value of the AHI, OSA severity can be categorized as mild (5 events/h ≤ AHI < 15 events/h), moderate (15 events/h ≤ AHI < 30 events/h), or severe (AHI ≥ 30 events/h) (13).

OSA is driven by multiple factors. Obesity is the most important modifiable risk factor, and the prevalence of OSA in obese individuals can be as high as 40 to 80% (14). Excessive fat accumulation, especially in the neck and upper respiratory tract, can contribute to airway narrowing and increases the risk of airway collapse during sleep. Aging is significantly associated with an increased prevalence of OSA, with a prevalence approaching 50% in older adults and increasing further among adults aged >80 years (15). The prevalence of OSA in men is approximately 2–3 times higher than that in women (16). However, the prevalence of OSA in postmenopausal women increases approximately twofold and is comparable to that in men (17), which may suggest a protective effect of sex hormones. Abnormalities in craniofacial structures, such as mandibular retraction, micrognathia, and tonsil hypertrophy, can increase the risk of airway collapse by reducing the bone-like framework of the pharyngeal region (18). In addition, smoking and alcohol consumption can exacerbate OSA severity. Smoking increases airway resistance through upper airway inflammation and edema (19), whereas alcohol consumption exacerbates nocturnal respiratory events by increasing upper airway resistance or changing the arousal threshold (20).

## 3 Structure and function of the GS

In 2012, Iliff and Nedergaard from the University of Rochester Medical Center in the United States described fluid exchange between cerebrospinal fluid (CSF) and interstitial fluid in mice, and named it the glial-lymphatic system. Because of its similarities to the peripheral lymphatic system, it is also known as the “glymphatic system” (GS) (4). This finding challenged the traditional understanding that the central nervous system lacks lymphoid clearance pathways. The GS consists of three core parts: periarterial CSF inflow pathways, exchange between CSF and interstitial fluid in the brain parenchyma, and perivenous clearance pathways (21). The GS is a whole-brain fluid transport network around blood vessels. CSF enters the brain parenchyma from the subarachnoid space along the periarterial space, mainly driven by the transmural pressure gradient generated by arterial pulsation. Aquaporin-4 (AQP4), which is densely expressed in astrocytes, plays a key role in this process by mediating the movement of CSF across the astrocytic end foot membrane into the brain interstitial space. After entering the brain parenchyma, the CSF mixes with the interstitial fluid, carrying metabolic waste including β-amyloid (Aβ), and then flows along the perivenous space, and finally being discharged from the cranial cavity through the cervical lymphatic system and meningeal lymphatic vessels (22–27). It is essential to note that the GS and meningeal lymphatic system are anatomically located in different compartments; however, they are functionally connected. After the GS transports waste within brain tissue, it channels metabolites into the meningeal lymphatic system. This system then transports them to the cervical lymph nodes for final clearance. The integrity of this series of connections is a crucial step in understanding how OSA may contribute to EPVS formation via GS dysfunction.

The GS is not only a core pathway for clearing metabolic waste in the brain but also participates in various important physiological processes. In addition to removing potential neurotoxic metabolites such as Aβ and lactic acid, the GS may contribute to the transport of glucose, lipids, amino acids, growth factors, and neuromodulators from the CSF to various regions of the brain parenchyma (28). GS activity shows significant circadian changes, and its clearance efficiency is increased during sleep, especially non-rapid eye movement (NREM) sleep. Animal experiments have shown that the clearance efficiency of the GS during sleep is approximately 60% higher than during wakefulness (29). GS dysfunction can reduce the clearance of metabolic waste, potentially contributing to neurological inflammation and dysfunction.

## 4 Definition and imaging characteristics of PVS

PVS are fluid-filled spaces surrounding the small arteries, microarteries, capillaries, microveins, and small veins in the brain that extend along the blood vessels from the brain surface into the brain parenchyma. The Virchow–Robin spaces (30) was standardized as PVS in the 2013 Neuroimaging Vascular Changes Reporting Standard (STRIVE-1) (31). In 2023, the STRIVE working group published an updated version of the standard (STRIVE-2), further clarifying the imaging definition and evaluation criteria of the PVS (32). According to the STRIVE-2 criteria, on routine MRI, the PVS appear as well-defined circular, oval, or linear structures with signal intensity similar to that of CSF; that is, high signal on T2-weighted imaging (T2WI) and low signal on T1-weighted imaging (T1WI), fluid-attenuated inversion recovery (FLAIR), and diffusion-weighted imaging (DWI) without restricting diffusion. PVS are commonly found in the basal ganglia (BG), centrum semiovale (CSO), and midbrain, with a diameter usually no greater than 2 mm (measured perpendicular to the blood vessel) and are mostly 1–2 mm in diameter. When they enlarge to a certain extent (>0.66 mm), it may become visible on MRI (5, 32). Specifically, STRIVE-2 noted that the PVS should not be confused with isolated developmental vascular structures in the lower BG region (32).

In addition, the PVS load is often semiquantitatively evaluated using the Potter scale (5, 33). This scale uses the cerebral hemisphere with the greater PVS burden and grades PVS of the BG and CSO, whereas different grading criteria are used for the midbrain (Table 1).

**Table 1:** PVS classification criteria for different anatomical sites on the Potter scale.

Grade	Number of PVS in BG/CSO	Number of PVS in midbrain
0	0	No PVS visible
1	1–10	PVS visible
2	11–20	–
3	21–40	–
4	>40	–

## 5 Association between GS dysfunction and EPVS

There is a bidirectional relationship between the decline in GS function and EPVS. A decrease in GS function leads to impaired metabolic waste clearance and poor drainage of interstitial fluid and may cause passive expansion of the PVS. However, after PVS expansion, fluid retention around the blood vessels may further impair the exchange of CSF and interstitial fluid, forming a vicious cycle (34). Animal experiments have shown that brain GS dysfunction is closely associated with an increase in EPVS (35). Similarly, in humans, EPVS severity is negatively correlated with Aβ clearance from the CSF and positively correlated with brain Aβ deposition (36). Together, these findings support the use of EPVS as a non-invasive imaging marker of impaired GS function (37). STRIVE-2 further supports this view at the international consensus level, indicating that PVS visible on MRI may reflect impaired clearance of interstitial fluid and metabolic waste in the brain, and that the clearance process is enhanced during sleep. The core pathological feature of OSA is the disruption of sleep architecture (in particular, slow-wave sleep reduction and sleep fragmentation). Therefore, the STRIVE-2 consensus provides atheoretical link between sleep and clearance function and provides an authoritative theoretical basis for the hypothesis that OSA-related sleep fragmentation may contribute to GS dysfunction and thereby promote EPVS formation (32).

## 6 Pathophysiology of OSA-related GS dysfunction and EPVS formation

There are four main mechanisms by which OSA damages GS function and promotes EPVS: sleep structure disruption, intermittent hypoxia, hemodynamic abnormalities, and BBB damage.

### 6.1 Sleep structure damage

Patients with OSA experience reduced slow-wave sleep, fragmented sleep, and frequent microarousals. Animal experiments have shown that clearance efficiency during sleep is approximately 60% higher than during wakefulness (38). Clinical data suggest that slow-wave sleep duration is negatively correlated with CSF Aβ levels, suggesting that reduced slow-wave sleep may impair the clearance capacity of Aβ (39). Even if the total sleep time remains unchanged, fragmentation itself disrupts slow-wave continuity, affecting GS function and potentially contributing to EPVS formation. In mouse experiments, long-term sleep deprivation reduced the efficiency of glymphatic clearance by about 40% (40).

### 6.2 Intermittent low oxygen

Intermittent hypoxia is a pathophysiological feature characteristic of OSA. It can activate hypoxia-inducible factor-1α (HIF-1α) and nuclear factor-κB (NF-κB) signaling pathways, which can contribute to oxidative stress and systemic and central inflammatory responses (41). Chronic intermittent hypoxia-driven pro-inflammatory responses can lead to infiltration of inflammatory cells into the PVS. This infiltration not only physically blocks fluid drainage but also may cause local tissue damage by releasing inflammatory mediators such as interleukin (IL)-1β, IL-6, and tumor necrosis factor (TNF)-α. In addition, at the molecular level, intermittent hypoxia can inhibit the expression and polarized distribution of AQP4in astrocytic foot processes, weakening the driving force of CSF flow. Together, these changes may lead to a decrease in GS clearance function and pathological expansion of the PVS (8, 42).

### 6.3 Hemodynamic abnormalities

In patients with OSA, the intrathoracic pressure fluctuates dramatically during sleep, dropping to −50 to −80 cmH_2_O during inhalation and rapidly rising to positive pressure during exhalation (43). This pressure change is transmitted to the brain, potentially causing fluctuations in intracranial pressure, cerebral blood flow oscillations, and disturbances in CSF pulsation. The flow of CSF along the peripheral space is influenced by the pulsation of the arteries, which is the core driving force for GS convective transport (44, 45). Patients with OSA often have hypertension, and long-term poor blood pressure control can lead to small-vessel arteriosclerosis and endothelial dysfunction, increased cerebral arterial pulsation, and alterations in surrounding tissue integrity. Weakening of the pulsating driving force may impair GS function, whereas abnormally enhanced pulsating stress may contribute to expansion of the PVS, resulting in structural damage. In addition, blood oxygen–level dependent functional MRI (BOLD-fMRI)revealed abnormal changes in cerebrovascular reactivity (CVR) in patients with OSA, which manifested as a compensatory increase in overall CVR and impairment of regional vasodilatory function (46). This vasoconstrictive response may further exacerbate arterial pulsation abnormalities, which together may contribute to the hemodynamic mechanisms underlying of EPVS.

### 6.4 BBB damage

OSA-induced inflammation and oxidative stress may disrupt the BBB (47). After BBB damage, plasma components penetrate the brain parenchyma, potentially causing vascular edema and glial cell activation, which may interfere with the GS. Simultaneously, the loss of BBB integrity may allow fluid and inflammatory cells to leak into the PVS. This may increase the fluid load in the interstitial space and further contribute to the EPVS formation. The association between BBB damage and EPVS has been confirmed using imaging studies (48, 49).

In summary, the above four mechanisms do not exist in isolation, but may constitute a synergistic and amplified pathological network. There is a profound interaction between intermittent hypoxia and disruption of sleep architecture. Frequent microarousal caused by sleep fragmentation can disrupt neurovascular coupling synchronized with slow-wave sleep, directly weakening the driving effect of arterial pulsation on the flow of CSF into the PVS. At the same time, inflammatory factors released by inflammatory pathway activated by intermittent hypoxia may impair the polarized distribution and function of AQP4 in astrocytes and exacerbate BBB damage. These two core mechanisms reinforce each other: sleep disruption may aggravate hypoxic stress, and inflammatory reactions further interfere with sleep homeostasis, forming a vicious cycle that damages GS clearance and promotes EPVS. Therefore, GS dysfunction is a unified pathological pathway connecting sleep architecture abnormalities, hypoxia, hemodynamic changes, and BBB damage, and is also a key intermediary in OSA-related EPVS (Figure 1).

**Figure 1:**
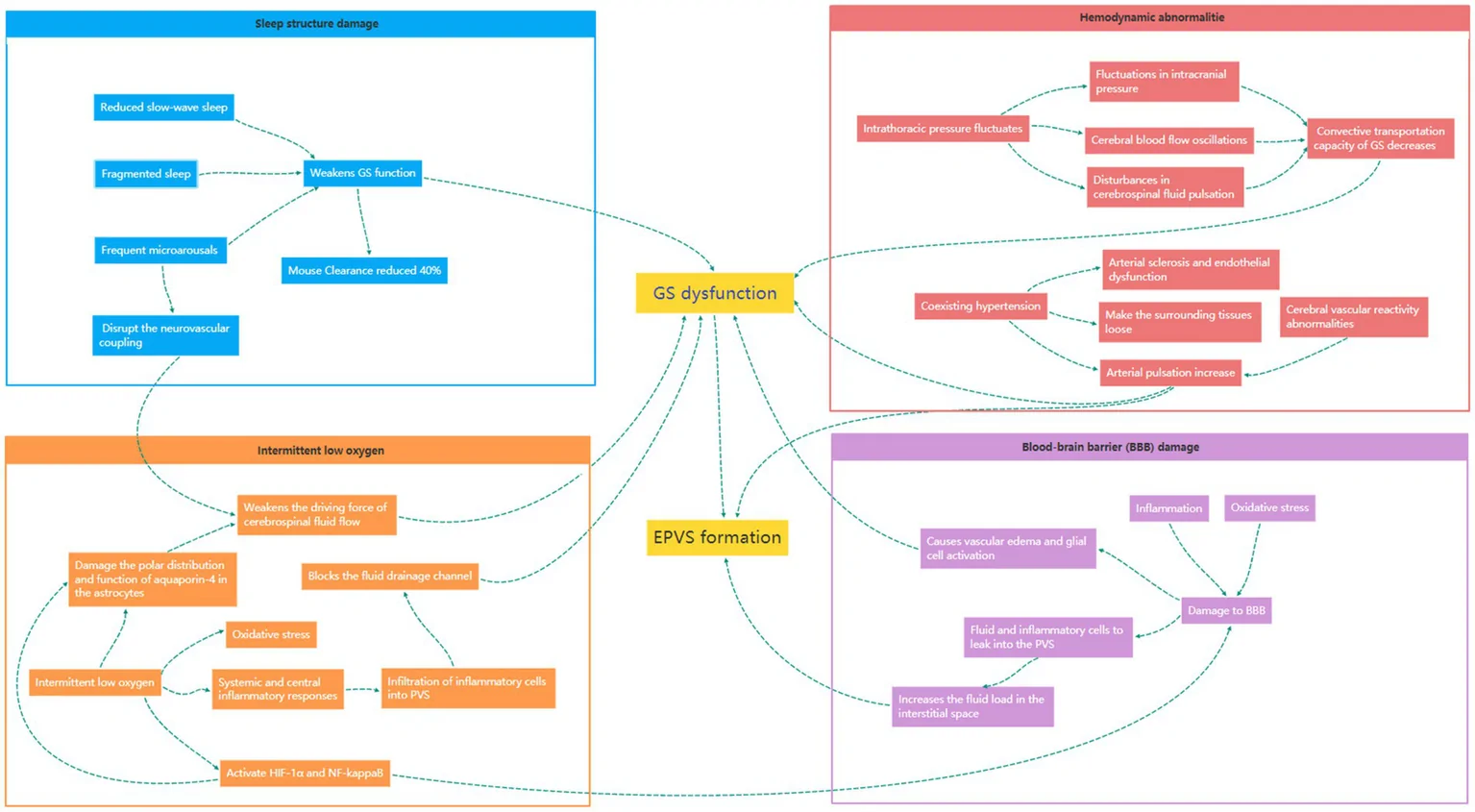
Pathophysiological mechanism of OSA-related GS dysfunction and EPVS formation. OSA, obstructive sleep apnea; GS, glymphatic system; EPVS, enlarged perivascular spaces.

## 7 Multimodal MRI assessment of OSA and EPVS

### 7.1 Routine MRI: sleep structure and regional distribution of EPVS

Existing imaging studies have confirmed that OSA is closely related to increased EPVS burden and that this association exhibits significant regional specificity. The main distribution sites of EPVS in patients with OSA are the BG and CSO (32), and OSA severity (measured by AHI) is positively correlated with EPVS grade or volume in these areas (50, 51). Further research found that the BG-EPVS burden was independently associated with cognitive impairment in patients with OSA, suggesting that this region may be a key structural basis for OSA-related cognitive decline (52). These findings suggest that moderate-to-severe OSA can promote the development of EPVS. This may further worsen cognitive impairment.

In addition to OSA severity, disruption of sleep architecture is another important mechanism that connects OSA to EPVS. Multiple studies have shown that sleep architecture abnormalities in OSA and decreased sleep efficiency may contribute to the development of EPVS (53). Sleep architecture abnormalities may not only affect GS clearance, but also be associated with CSVD, providing a potential mechanism for the biological link between sleep disorders and CSVD (54). The typical changes in sleep structure in patients with OSA mainly manifest as slow-wave sleep (N3), decreased REM sleep, increased N2 sleep proportion, increased arousal index, and decreased sleep efficiency. MRI studies have confirmed that these changes in sleep parameters are closely related to EPVS; however, the current findings are inconsistent. Baril et al. (54) found that the proportion of N3 sleep was negatively correlated with CSO-EPVS. However, no significant associations were observed between total sleep time, sleep efficiency, N2 and REM sleep ratios, AHI, or CSO-EPVS. In contrast, Jia et al. (55) showed that the lowest blood-oxygen saturation, N3 stage, and REM sleep ratio were negatively correlated with total EPVS, whereas the awakening index and N2 stage sleep ratio were positively correlated with total EPVS. Other studies have reported a negative correlation between sleep efficiency and total EPVS and BG-EPVS, and between the N3 phase and BG-EPVS (56). These findings suggest that OSA-induced sleep fragmentation and deep sleep deprivation may contribute to EPVS formation by impairing nighttime GS clearance. The heterogeneity of the study results may be related to differences in the distribution of OSA severity, age distribution, EPVS assessment methods, and control for confounding factors in the study populations. A decrease in the proportion of REM sleep has been shown to be an independent risk factor for moderate-to-severe BG-EPVS (32), suggesting that improving REM sleep quality and sleep efficiency may play an important role in preventing BG-EPVS progression. Moreover, Sommer et al. (57) found in a longitudinal observational study of continuous positive airway pressure (CPAP) treatment that patients with OSA who had good adherence (≥4 h/night on≥70% nights) maintained stable BG-PVS volume, whereas those with poor adherence showed a significant increase in BG-PVS volume, suggesting that optimal CPAP use may slow the progression of EPVS.

### 7.2 Voxel-based morphological analysis

Voxel-based morphological analysis (VBM) is a whole-brain, automated, and unbiased quantitative analytical method performed at the voxel level. It can non-invasively calculate changes in brain gray matter volume, density, or concentration (58). VBM can reveal morphological changes in the brain tissues of patients with OSA at the voxel level and is widely used in the field of neuroimaging. In patients with OSA, a VBM study revealed widespread gray matter atrophy, mainly located in areas critical for cognition, including the frontal lobe, cingulate gyrus, temporal cortex, and cerebellum (48, 49, 59, 60). In addition, a study of children with OSA found that the volume of gray matter in the right middle frontal gyrus was larger than that in children without OSA and was negatively correlated with the expressive attention score (61). Although these studies did not directly measure the PVS, they demonstrated that OSA causes structural damage to brain tissue. Such tissue damage may alter the adjacent microenvironment and promote the expansion of the peripheral PVS and the progression of cognitive impairment.

### 7.3 Diffusion tensor imaging

Diffusion tensor imaging (DTI) is an MRI technique based on the principle of the diffusion of water molecules in a Gaussian distribution. By applying diffusion-sensitive gradient fields in multiple directions, the degree and rate of diffusion of water molecules in biological tissues in different directions can be quantitatively analyzed, thereby providing information on the integrity of the white matter microstructure. This is a powerful tool for studying early brain dysfunction in patients with OSA (62). Key indicators include fractional anisotropy (FA, a measure of directional consistency), mean diffusivity (MD, a measure of overall diffusion), axial diffusivity (AD, related to axon integrity), and radial diffusivity (RD, related to myelination) (63, 64). In patients with OSA, DTI has demonstrated that FA decreases and MD/RD increases in a wide range of white matter areas, including the corpus callosum, internal capsule, frontal white matter, and areas that appear normal on routine MRI (65, 66). These changes suggest demyelination, axon loss, and increased tissue water content, which are consistent with chronic hypoxic–ischemic dysfunction. Another study based on tract-based spatial statistics analysis also supported this view, showing a significant correlation between the FA and MD values of the corpus callosum and thalamic tracts and the overall cognitive ability of patients with CSVD (67). This indicates that patients with OSA have pathological changes closely related to cerebral small-vessel lesions. OSA can cause diffuse cerebral small-vessel disease and BBB disruption via hypoxia, inflammation, and endothelial dysfunction. The overall degree of white matter microstructural damage in patients with OSA is associated with EPVS burden (68). This indicates that EPVS and DTI abnormalities occur at different levels during the same pathological process. The extensive white matter damage found on DTI provides a pathophysiological context for EPVS, and OSA may contribute to extensive damage to cerebral small blood vessels and brain parenchyma.

### 7.4 DTI analysis along the PVS

In 2022, Taoka et al. (69) proposed a non-invasive DTI analysis along the PVS (DTI-ALPS) based on DTI to assess GS function in the human brain. The ALPS index was calculated by comparing the diffusivity of water molecules in the direction of the PVS with that along the projection of fibers adjacent to the lateral ventricles and the association fibers perpendicular to this direction. A lower ALPS index reflects a decrease in fluid diffusion around the vessel, suggesting impaired GS function (60). Several preliminary studies have explored the value of the DTI-ALPS in patients with OSA. A meta-analysis of patients with OSA suggested that severe OSA is associated with impaired lymphatic activity, and the DTI-ALPS index may be a tool for assessing OSA-related GS dysfunction (70). Multiple studies have shown a significant decrease in the ALPS index in patients with OSA compared with healthy controls, providing preliminary imaging evidence for OSA-related GS dysfunction (51, 71, 72). Lee et al. (73) found that OSA severity in patients was associated with the DTI-ALPS index and memory changes, and that OSA may be associated with cognitive impairment through perivascular fluid dynamics, which may be particularly related to disruption of specific sleep stages (e.g., NREM sleep periods) (74). This supports the hypothesis that OSA-related sleep fragmentation may impair sleep-dependent GS function. Other studies have found that the ALPS index may mediate the relationship between the EPVS load and cognitive performance, suggesting that GS dysfunction is a key intermediate step in the association between EPVS and cognitive decline (75, 76). Additionally, the ALPS index is associated with memory function in patients with OSA, further suggesting its clinical relevance (77). Some studies have also reported a negative correlation between the ALPS index and EPVS scores (70), providing evidence consistent with the hypothesis that EPVS is a morphological manifestation of impaired GS function.

However, the correlation between DTI-ALPS and the gold standard for GS function remains controversial. Mossige et al. (78) directly compared DTI-ALPS with intrathecal contrast-enhanced MRI, and found that the ALPS index was negatively correlated with deep white matter 24-h tracer enrichment. However, the concentration of the tracer in the ALPS region of interest was only 10% (up to 68–81% in the cortical layer), and there was no significant correlation with the 48-h tracer kinetics. These results suggest that the ALPS index may mainly reflect perivascular diffusion in specific areas (e.g., paraventricular) and has limited correlation with the overall assessment of whole-brain GS function. Therefore, the results of DTI-ALPS alone should be interpreted with caution when assessing GS function in patients with OSA. In recent years, research on the combined application of PVS volume fraction and the DTI-ALPS index to evaluate the function of the GS has emerged (79, 80). The PVS volume fraction reflects the severity of EPVS and is a structural index of the GS. The DTI-ALPS index indirectly provides an indirect measure of perivascular water diffusivity around blood vessels and is a functional indicator. The combination of the two can form a “structure–function” complementary evaluation system, which helps to distinguish between simple EPVS and GS dysfunction-associated EPVS, and reveals the heterogeneity of GS dysfunction in patients with OSA.

### 7.5 Diffusion kurtosis imaging

Diffusion kurtosis imaging (DKI) is an MRI technique based on the non-Gaussian diffusion of water molecules, which provides a more sensitive measurement of tissue microstructure complexity by using higher b-values to characterize the diffusion heterogeneity of brain tissue (81, 82). The mean kurtosis (MK) value reflects the complexity of the tissue microstructure (83). Studies of OSA have suggested that DKI parameters, especially MK values, may be more sensitive than DTI parameters for detecting subtle white matter changes (84). Lower MK values in the white matter of patients with OSA may reflect tissue degeneration and reduced structural complexity, which may precede or accompany the development of EPVS (85). Li et al. (66) found through whole-brain DKI and DTI analyses that the kurtosis anisotropy and FA values in the white matter of children with OSA were significantly reduced, with kurtosis anisotropy showing a wider range of changes. These results indicate that DKI can more sensitively detect damage to the microstructure of brain tissue in patients with OSA and that there is a correlation between abnormal changes in the microstructure of some brain regions and cognitive dysfunction.

### 7.6 Dynamic contrast-enhanced MRI

Dynamic contrast-enhanced (DCE) MRI is a functional imaging technique that uses rapid repeated scanning to monitor the distribution and clearance of contrast agent in tissues after the intravenous injection of a gadolinium-based contrast agent. This technique quantitatively evaluates the BBB permeability and tissue perfusion characteristics by calculating pharmacokinetic parameters such as the transfer constant (K_trans_) for contrast-agent leakage from the vascular lumen to the extravascular extracellular space, extravascular extracellular space volume fraction (Ve), and plasma volume fraction (Vp) (86, 87). In OSA studies, the application of DCE-MRI primarily focuses on assessing BBB function. Wang et al. (88) found that the K_trans_ values in the hippocampus and temporal cortex of patients with severe OSA were significantly increased, indicating increased BBB permeability, and were negatively correlated with cognitive function scores. This study further revealed that a reduction in the fluid washout rate in the perivascular space is closely related to BBB damage. This suggests that GS drainage function is impaired and that this functional impairment is closely related to EPVS and cognitive decline. Moreover, after 1 month of CPAP treatment, patients with OSA showed a significant improvement in glymphatic drainage function, suggesting that improved ventilation may help alleviate OSA-related GS dysfunction and thereby delay cognitive decline.

### 7.7 Phase-contrast MRI and 4D flow MRI

Phase-contrast (PC) MRI uses bipolar gradient fields to perform phase-coded imaging of flowing protons. A pair of gradient pulses with the same intensity and duration but opposite directions are applied in the flow direction, and the phase changes of the stationary tissue cancel each other out, whereas the residual phase difference is generated by the positional changes of the flowing protons. This phase difference can be used to distinguish blood flow from the surrounding tissue and calculate the flow rate (89, 90). 2D PC-MRI, as a conventional PC technique, can directly measure the flow velocity of CSF in the aqueductal and subarachnoid spaces, providing a reference for evaluating the driving force of the GS (91). 4D flow MRI further provides parameters such as 3D whole-brain velocity fields, vorticity, and kinetic energy (92).

In terms of the driving mechanism of GS, the current literature mainly provides general physiological conclusions based on healthy populations or fluid mechanics models. The synergy between arterial pulsation and fluctuations in transcerebral parenchymal pressure is the key mechanism driving the directional flow of CSF within the interstitial spaces surrounding blood vessels (93). Respiratory movements are also an important factor influencing the overall flow of CSF throughout the brain (94). When extending these mechanisms to OSA research, it is necessary to distinguish between the reported OSA-specific evidence and the inferences based on physiological mechanisms. Currently, there are still few studies that directly use PC-MRI or 4D flow MRI to assess changes in CSF pulsation in patients with OSA. The existing evidence specific to OSA mainly comes from studies of CVR, suggesting that patients with OSA have hemodynamic abnormalities and increased vascular stiffness (95). Based on this, OSA may indirectly affect the pulsation of CSF, but this association still needs to be verified through direct measurement using PC-MRI or 4D flow MRI. Similarly, intermittent hypoxia and marked fluctuations in intrathoracic pressure characteristic of OSA may interfere with the CSF flow driven by respiration (94). This inference has an important pathophysiological basis, but there is currently a lack of imaging evidence in patients with OSA. In summary, the advantages of PC-MRI and 4D flow MRI lie in their ability to directly assess the macroscopic fluid dynamics of CSF, and they can complement the DTI-ALPS index.

### 7.8 BOLD-fMRI

Based on the paramagnetic effect of deoxygenated hemoglobin, BOLD-fMRI indirectly reflects the vascular response by detecting changes in the blood oxygen level in local brain tissue. In recent years, this technology has been widely used for the quantitative assessment of CVR. CVR refers to the ability of the cerebrovascular system to respond to vascular activity stimulation (such as increased CO_2_) and is an important physiological indicator that reflects the reserve function of small blood vessels in the brain. BOLD-fMRI can non-invasively evaluate whole-brain and regional CVR through breath-holding tasks or exogenous CO_2_ inhalation paradigms. Its core quantitative evaluation indicators mainly include the CVR amplitude (% BOLD·mmHg^−1^) which reflects vascular reserve capacity, and the time to peak, which reflects the timing and speed of the hemodynamic response. In patients with OSA, BOLD-fMRI has revealed complex changes in CVR. Dehnavi et al. (46) found in a systematic review and meta-analysis that the overall CVR of patients with OSA was higher than that of the control group, and with CVR in the gray matter and white matter significantly enhanced. This result is consistent with the findings of Wu et al. (96) using a breath-holding task and may reflect the compensatory vascular regulation mechanism of brain tissue under intermittent hypoxia exposure. There is a potential pathophysiological association between CVR abnormalities and EPVS. Kapoor et al. (97) found a significant negative correlation between the PVS load and whole-brain CVR in an elderly population. A longitudinal study by Libecap et al. (98) further confirmed that low CVR in the BG region at baseline predicts an increase in the number of EPVS in this region after approximately 2.5 years, which remained significant after controlling for age, sex, baseline EPVS, and risk factors for CSVD. These findings suggest that abnormalities in CVR detected by BOLD-fMRI are a key functional link connecting OSA-related hemodynamic disfunction and EPVS formation. OSA induces impaired CVR, exacerbating arterial pulsation abnormalities and thereby promoting pathological EPVS.

The various MRI techniques mentioned do not replace each other but form a multimodal complementary pattern from structure to function and from microstructural damage to hemodynamic assessment, providing multidimensional imaging evidence for OSA-related GS dysfunction and EPVS formation. The key measurement parameters, advantages, and limitations of each technology are summarized in Table 2.

**Table 2:** Application of various MRI techniques in the evaluation of OSA-related GS dysfunction and EPVS.

Technique	Summary of the technique	Measured parameter	Key OSA/EPVS finding	Limitations
Routine MRI	PVS of BG and CSO visually graded based on routinestructural images such as T1, T2, and FLAIR.	Potter scale	Patients with OSA have an increased burden of EPVS, primarily distributed in the BG and CSO, and this is associated with the OSA severity as measured by the AHI; BG-EPVS burden is independently associated with cognitive impairment. Sleep fragmentation, reduced N3 and REM sleep are associated with an increase in EPVS, but there is heterogeneity among studies.	Semiquantitative evaluation, strong subjectivity; GS functional status cannot be determined
VBM	Using high-resolution T1-weighted images, whole-brain gray matter volume/density is quantified automatically and unbiased at the voxel level.	Gray matter volume/density	Patients with OSA exhibit widespread gray matter atrophy, primarily affecting the frontal lobe, cingulate gyrus, temporal cortex, and cerebellum. In children with OSA, the gray matter volume in the right middle frontal gyrus is increased and correlates with attention scores. These structural changes may alter the perivascular microenvironment, promoting EPVS.	PVS is not directly measured; GS function cannot be evaluated
DTI	Based on the Gaussian diffusion model of water molecules, the integrity of white matter microstructure is quantified through multi-directional diffusion-sensitive gradients.	FA, MD, AD, RD	In patients with OSA, extensive white matter area (corpus callosum, inner capsule, frontal white matter) shows decreased FA and increased MD/RD, indicating demyelination and axon loss. The degree of overall white matter damage is related to EPVS load, suggesting a common small-vessel pathology basis.	Gaussian model limitations (cannot reflect non-Gaussian diffusion); GS function cannot be directly evaluated
DTI-ALPS	ALPS index is calculated by comparing diffusion rates in the perivascular direction with those in the vertical direction of the projection and association fiber regions of the lateral ventricular body, serving as an indirect indicator of GS function.	ALPS index	The ALPS index in OSA patients is significantly reduced, suggesting GS dysfunction; this index has been reported to mediate the relationship between EPVS load and cognitive performance and is associated with memory function; most studies show a negative correlation between the ALPS index and EPVS grading.	There is a dispute over the relevance to the GS functional gold standard. It mainly reflects the partial diffusion of the lateral ventricle and cannot represent whole-brain GS function.
DKI	Using higher b-values to simulate non-Gaussian diffusion of water molecules provides a more sensitive measurement of tissue microstructural complexity.	MK value, kurtosis anisotropy values	Patients with OSA exhibit reduced MK in white matter regions, indicating a loss of tissue structural complexity; in pediatric OSA patients, DKI can detect subtle changes more extensively than DTI, and these changes may precede or accompany EPVS and are associated with cognitive impairment.	Long scanning time, low clinical popularity, complex post-treatment
DCE-MRI	After intravenous injection of gadolinium contrast agent, rapid repeated T1-weighted imaging is performed to quantitatively analyze the process of contrast agent leakage from the vascular lumen into the brain parenchyma, thereby assessing BBB integrity.	K_trans_, Ve, Vp	In patients with severe OSA, elevated K_trans_ values in the hippocampus and temporal cortex suggest increased BBB permeability, which is associated with a decline in cognitive scores; reduced perivascular fluid clearance is related to BBB damage and progression of EPVS.	Invasive (injection of contrast agent is required); risks associated with contrast agents; long scan times
PC-MRI and 4D Flow MRI	PC-MRI (2D or 4D) uses bipolar gradients to encode the velocity of flowing protons; 4D flow provides a 3D time-resolved velocity field of blood flow and CSF, enabling the assessment of macroscopic fluid dynamics.	CSF flow rate, vorticity, kinetic energy	PC-MRI/4D flow can directly assess macroscopic CSF dynamics; however, changes in CSF flow velocity or pulsation in OSA have not been confirmed by direct measurement. Indirect evidence from CVR studies suggests that OSA-related hemodynamic abnormalities and increased vascular stiffness may affect CSF pulsation, and intermittent hypoxia and marked intrathoracic pressure fluctuations may interfere with respiratory-driven CSF flow; these hypotheses require direct PC-MRI or 4D flow validation.	High hardware requirements, complex post-processing, requires complementary evaluation of GS function using the ALPS index
BOLD-fMRI	Detects changes in local blood oxygenation and quantitatively assesses CVR by combining vascular activation (breath-holding or CO₂ inhalation).	Cerebrovascular reactivity amplitude (% BOLD·mmHg^−1^), peak time	Some studies have shown that patients with OSA exhibit an overall increase in CVR (compensatory vascular regulation) but reduced CVR in the BG region can predict an increase in future EPVS; abnormal CVR serves as a functional bridge between hemodynamic impairment of OSA and formation of EPVS.	GS function cannot be directly evaluated. Breath-holding task requires patient cooperation.

AD, axial diffusion coefficient; AHI, apnea–hypopnea index; ALPS, analysis along the perivascular space; BBB, blood–brain barrier; BG, basal ganglia; BOLD, blood oxygen level–dependent; CSF, cerebrospinal fluid; CSO, centrum semiovale; CVR, cerebrovascular reactivity; DKI, diffusion kurtosis imaging; DTI, diffusion tensor imaging; EPVS, enlarged perivascular spaces; FA, fractional anisotropy; FLAIR, fluid-attenuated inversion recovery; fMRI, functional magnetic resonance imaging; GS, glymphatic system; MD, mean diffusion coefficient; MK, mean kurtosis; MRI, magnetic resonance imaging; OSA, obstructive sleep apnea; PC, phase contrast; PVS, perivascular spaces; RD, radial diffusion coefficient; REM, rapid eye movement; VBM, voxel-based morphometry.

## 8 Summary and outlook

Multimodal MRI studies continue to demonstrate the close and complex association between OSA and EPVS. Intermittent hypoxia and fragmented sleep are the key drivers of this association, primarily through impaired GS function. Routine MRI has identified EPVS mainly in the BG and CSO, and EPVS burden is associated with specific abnormalities in sleep structures. New MRI technologies, such as DTI and its derivatives, can further deepen our understanding of this relationship. DTI reveals extensive white matter microstructural damage around the PVS, providing a pathophysiological context for EPVS. DTI-ALPS provides indirect non-invasive imaging evidence for GS dysfunction in patients with OSA, establishing its core intermediary position in the OSA–GS–EPVS pathological axis. DCE-MRI suggests that BBB damage may be related to a decline in GS drainage function and EPVS formation.

Current research is transitioning from exploring correlations to investigating potential causal mechanisms. Future research should focus on the following directions. First, in a large prospective longitudinal cohort, the EPVS burden (e.g., PVS volume fraction) should be accurately quantified, and the clinical robustness and reproducibility of functional indicators such as the DTI-ALPS index should be verified. Second, multimodal imaging technologies should be integrated (e.g., arterial spin labeling, 4D flow MRI, and fMRI) to construct a comprehensive model of OSA-related cerebral small-vessel disease, GS dysfunction, and neurological changes to identify high-risk populations with cognitive decline. Finally, future studies should explore imaging biomarkers, especially the combined use of EPVS burden and the DTI-ALPS index, to evaluate their role in assessing the efficacy of interventions (such as CPAP therapy). This approach may provide an objective tool for evaluating targeted treatment strategies aimed at preserving GS function and may reveal new imaging pathways for the early intervention for OSA-related brain injury. Existing studies suggest that CPAP treatment may have beneficial effects on the progression of EPVS, GS function, and cognitive function, but the relevant imaging evidence remains limited and requires further validation in longitudinal studies.
